# Linking Soil Microbial Diversity to Nitrogen and Phosphorus Dynamics

**DOI:** 10.3390/microorganisms13102401

**Published:** 2025-10-21

**Authors:** Bruna Arruda, Eduardo Mariano, Wilfrand Ferney Bejarano-Herrera, Fábio Prataviera, Elizabeth Mie Hashimoto, Fernando Ferrari Putti, Jéssica Pigatto de Queiroz Barcelos, Paulo Sergio Pavinato, Fernando Dini Andreote, Davey L. Jones

**Affiliations:** 1 Agronomy Engineering Program, University of Applied and Environmental Sciences (U.D.C.A), Bogotá 111166, Colombia; 2 Center for Nuclear Energy in Agriculture (CENA), University of São Paulo (USP), Piracicaba 13416-000, Brazil; emariano@cena.usp.br; 3 Colombian Corporation for Agricultural Research (AGROSAVIA), Pasto 520038, Colombia; wbejarano@agrosavia.co; 4 “Luiz de Queiroz” College of Agriculture (Esalq), University of São Paulo (USP), Piracicaba 13418-900, Brazil; fabio_prataviera@usp.br (F.P.); pavinato@usp.br (P.S.P.); fdandreo@usp.br (F.D.A.); 5 Academic Department of Mathematics, Federal University of Technology, Londrina 86036-700, Brazil; ehashimoto@professores.utfpr.edu.br; 6 Biosystems Engineering Department, São Paulo State University (UNESP), Tupã 17602-496, Brazil; fernando.putti@unesp.br; 7 Department of Agronomy, University of Western São Paulo (UNOESTE), Presidente Prudente 19067-175, Brazil; jessicabarcelos@unoeste.br; 8 Environment Centre Wales, Bangor University, Bangor LL57 2UW, UK; d.jones@bangor.ac.uk

**Keywords:** diversity extinction, ^14^CO_2_ respiration, ammonium, nitrate, ^33^P lability

## Abstract

Changes in the soil microbial community for studies of different novel communities can be promoted by different methodologies, among which soil autoclaving stands out as a quick and readily available tool. However, this procedure may also directly or indirectly alter nitrogen (N) and phosphorus (P) dynamics. The purposes of this study were as follows: (i) to characterize microbial activity after soil autoclaving through microbial ^14^CO_2_-respiration; and (ii) to evaluate the effect of microbial manipulation and autoclaving on soil N and ^33^P dynamics. For this, two sets of soil samples from two areas (forest and cultivated area) were used in the laboratory. Firstly, ^14^C-glucose was added to the soils and after 24 h five soil microbiomes were generated: AS (autoclaved soil), and AS re-inoculated with serial dilutions (*w*/*v*) prepared by successive mixing of soil suspensions in sterile deionized water obtaining 10^−1^, 10^−3^, and 10^−6^, which generated the treatments AS + 10^−1^, AS + 10^−3^, and AS + 10^−6^; and the treatment NS (non-autoclaved control), all incubated for 28 d. ^14^CO_2_ emission was used to characterize microbial activity; additionally, N dynamics were assessed at the end of incubation. In a second assay, ^33^P was applied to the soil before autoclaving and re-inoculation. Following further incubation (14 d), a ^33^P chemical fractionation was performed. The following are based on the results: (i) ^14^CO_2_ emission: microbial activity in the autoclaved soil is null, but after a reinoculation of AS + 10^−1^ and AS + 10^−3^ soil dilution suspension, the ^14^CO_2_-respiration is higher than in an NS. (ii) regarding the N dynamics, in autoclaved soils, the microbial levels increased N-NH_4_^+^ concentration, with an evident increase in the AS + 10^−3^ and AS + 10^−1^, and a reduction in the N-NO_3_^−^ concentration in comparison to the NS. For ^33^P, the autoclaving procedure itself reduced the ^33^P lability, regardless of the levels of microbial community reinoculated.

## 1. Introduction

Soil micro-organisms play essential roles in carbon (C), nitrogen (N), and phosphorus (P) biogeochemical cycling. For C, cellulose represents the most abundant biopolymer in the plant–soil system [1], and its extracellular breakdown produces soluble products, such as cellobiose and glucose, which then become available for microbial uptake. In the N cycle, micro-organisms perform roles in biological N fixation, mineralization, nitrification, and denitrification [2]. The biodynamics of P include the immobilization and mineralization of organic P [3,4].

The distinct functions performed by the soil microbiota will depend on the diversity, abundance, and activity of the present organisms; hence, the soil microbial composition strongly depends on the plant effect, shaped by the rhizosphere [5]. Less-disturbed systems, such as natural forests, with high plant diversity and a high level of spatial heterogeneity, tend to have an equilibrium status and microbial stability [6]. On the other hand, climate change and anthropogenic activities such as agriculture (e.g., fertilization, tillage, and monocropping) induce shifts in the microbial community and trend to a reduction in biodiversity, mainly under monocultures, leading to a restriction in biodiversity [7]. Furthermore, management practices will affect the level of alterations in microbial community composition, which can either maintain biodiversity or strictly eliminate microbial communities, thereby altering the functioning of the soil system. In this sense, regenerative agriculture has been promoted as an ecological strategy to minimize the adverse effects of agriculture on the soil microbial diversity [8].

To explore the effects of different novel communities under controlled conditions, various methodologies, such as selective heat, specific biocides, dilution-to-extinction, and genome editing, can be employed, involving the loss of organisms and the subsequent reconstruction of the microbial community. These approaches simulate various levels of biodiversity, reflecting scenarios caused by management practices that can lead to biodiversity loss, and resulting in treatments that vary in community composition and function. Autoclaving is an option, as this process is rapid, low-cost, the equipment is usually part of the facilities in most microbiology laboratories, and no chemical pollution is generated [9]. This technique, which combines elevated temperature and pressure, causes the death of most of the organisms. From this sterile community, the recreation of the microbial community is possible by reincorporating diverse levels of biodiversity, reshaping the microbial community, and creating niches [10].

Once the soil microbial community is set, techniques to characterize the soil microbial changes and activity are required. Arruda et al. [11] used the autoclaving procedure to manipulate the soil micro-organisms and to identify the autoclaving effect on microbial community; the DNA sequencing procedure was used. These authors reported that both non-autoclaved and autoclaved soil had similar microbial diversity, for both fungi and bacteria, and attributed this result to the relic DNA. Therefore, DNA sequencing did not prove to be enough to identify changes in microbial activity after soil autoclaving. So, studies that aim to identify the changes in microbial activity caused by the autoclaving procedures are required. An alternative procedure for evaluating the soil microbial activity after autoclaving is to use labeled elements, such as C. As glucose is the most abundant and easily broken down in the soil system, ^14^C-glucose is highly recommended to track the C cycling, through the ^14^CO_2_ evolution from the soil, which reflects microbial respiration as a model substrate for understanding the priming effect in the soil and allows a correspondence of this process with the microbial activity [12].

However, the use of the autoclaving procedure has been questioned when the purpose of the study is to evaluate nutrient dynamics, as the autoclaving may affect the availability of some nutrients, such as N and P, either directly through the autoclaving conditions or indirectly through the loss of micro-organisms that participate in the nutrient cycling and availability. Therefore, the use of labeled elements, such as ^33^P, is an alternative to track the element in the soil and its dynamics and availability due to the autoclaving procedure.

The aims of this study were as follows: (i) to characterize microbial activity through microbial ^14^CO_2_-respiration after soil autoclaving; and (ii) to evaluate the effect of microbial manipulation and of the autoclaving procedure itself on soil N and ^33^P dynamics.

## 2. Materials and Methods

### 2.1. Soil Description

The soil, Eutric Cambisol, classified in previous studies [13] (Table 1), was sampled (0–10 cm depth and 1 m^2^—1 m × 1 m area) in the University of Wales Henfaes Experimental Station, Abergwyngregyn, Gwynedd, Wales, UK, during Autumn season, from a 24-year-old undisturbed Sycamore (*Acer pseudoplatanus*) plantation (53°14′22.70″ N, 4°0′54.81″ W) (named the forest area) and an adjacent cultivated area growing maize (*Zea mays* L.) (53°14′19.94″ N, 4°0′53.71″ W) (named the cultivated area). After sampling, the soil samples were homogenized individually and sieved (5 mm) to remove visible roots and stones, but we maintained the aggregates’ structure to keep the representativeness of the field conditions. The soil samples were stored in plastic bags and transported to the Bangor University facilities in a cooler to maintain the soil biological activity, and they were stored (4 °C) until the laboratory experiments were set up.

Additionally, four replicates of each soil (forest and cultivated) were sampled for chemical characterization. Soil samples were analyzed for chemical parameters such as pH and cation exchange capacity, macronutrients (N, P, K, Ca, Mg, and S) and micronutrients (Mn, Cu, B, Zn, Mo, and Fe) for the plant requirements using the following methodologies: pH was determined by water extraction and measured with a pH electrode/meter; P was measured by the Olsen method (sodium hydrogen carbonate extraction) and determined by solution spectrophotometry after complexing with ammonium molybdate; K by 1 M ammonium nitrate extraction and flame emission spectrometry (or Inductively Coupled Plasma—ICP) determination. Mg and Ca were extracted using 1 M ammonium nitrate, followed by atomic absorption spectrometry (or ICP). S was measured after calcium tetrahydrogen extraction and solution spectrophotometry of diorthophosphate-precipitated barium sulfate (or ICP). Mn was extracted with 1 M ammonium acetate containing 2 g L^−1^ quinol and analyzed by atomic absorption spectrometry (or ICP). Cu, Zn, and Fe were extracted with 0.05 M EDTA disodium salt and analyzed by atomic absorption spectrometry (or ICP). B was extracted by hot water extraction (80 °C) and determined by solution spectrophotometry after complexing with azomethine (or ICP). Mo was extracted with ammonium acetate (24.9 g L^−1^) and oxalic acid (12.6 g L^−1^) and analyzed by atomic absorption spectrometry with nitrous oxide (or ICP). Cation exchange capacity (CEC) was leached with 1 M ammonium acetate following a water rinse and determined by atomic absorption (or ICP).

### 2.2. Laboratory Assays—^14^C and N

**Figure 1:**
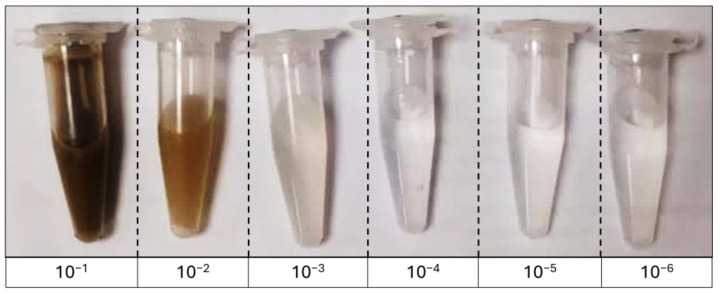
Scheme of dilution inoculum to be used in the diversity extinction method. Source: the authors (B.A. and W.F.B.H).

Firstly, for the ^14^C and N assay, 20 replicates of soil samples (5 g) were placed in individual sterile 50 mL polypropylene centrifuge tubes. Subsequently, 20 µM D-[U-14C]-glucose (Perkin Elmer Inc., Beaconsfield, UK, 250 µL; 6.8 kBq mL^−1^) was applied to the soil, and a trap with 1 M NaOH (1 mL) was placed above the soil surface [14]. The traps were sampled and replaced 2, 4, 8, and 24 h after ^14^C-glucose application. After 24 h, the ^14^C-treated soil replicates were used to generate five soil microbiomes with four replicates each. For this, 16 replicates were submitted to autoclaving (121 °C; 103 kPa; 1 h) to obtain the treatments: autoclaved soil (AS), as negative control (4 replicates), and AS re-inoculated with the treatments: (i) AS + 10^−1^ (4 replicates); (ii) AS + 10^−3^ (4 replicates); and (iii) AS + 10^−6^ (4 replicates). To obtain the serial dilutions, an undisturbed (4 °C) soil sample from each soil (forest or cultivated) was progressively reduced to the concentration of micro-organisms through successive mixing steps, respectively. For this, initially 100 mg of soil was taken and transferred to a 1.5 mL tube containing 900 µL of sterile deionized water, thus obtaining the 10^−1^ dilution (Figure 1). Subsequently, in the same way, 100 µL of this dilution was taken and transferred to another tube containing 900 µL of sterile deionized water, producing the 10^−2^ dilution, and so on until the serial dilution was reached (10^−6^). All the dilutions were homogenized (200 rpm, 1 h). Once we obtained the dilutions, 250 µL of soil suspension from the dilutions 10^−1^, 10^−3^, and 10^−6^ were obtained from NS and sterile deionized water in the AS. The positive control consisted of non-autoclaved soil (NS) (4 replicates). The treatments were incubated (room temperature at 20 ± 5 °C) [15].

Subsequently, ^14^CO_2_ emissions from the soil were monitored again by sampling the traps after 0.08, 0.17, 0.33, 1, 2, 3, 4, 5, 7, 10, 14, 18, 23, and 28 d after soil microbiome manipulation. The amount of ^14^CO_2_ trapped in the NaOH solution was determined by using 4 mL of Optiphase HiSafe 3 scintillation cocktail (PerkinElmer Inc., Waltham, MA, USA) in a Wallac 1404 liquid scintillation counter with automated quench correction (Wallac EG&G, Milton Keynes, UK). At the end, the soil was shaken with 0.5 M K_2_SO_4_ (25 mL; 200 rpm; 15 min). The extract was then centrifuged (18,000× *g*; 15 min), and the amount of N-NO_3_ and N-NH_4_ in the supernatant was determined colorimetrically using the methods of Miranda et al. [16] and Mulvaney [17], respectively. Also, ^14^C in the supernatant was determined using Optiphase HiSafe 3 scintillation cocktail (PerkinElmer Inc., Waltham, MA, USA) and a Wallac 1404 liquid scintillation counter with automated quench correction (Wallac EG&G, Milton Keynes, UK). The amount of ^14^C immobilized in the microbial biomass (^14^*Ci*) (kBq) was estimated by using Equation (1):

(1) C14i=C14a−C14O2+C14s

where ^14^*Ca* is the total ^14^C applied at the beginning of the experiment (kBq); ^14^*CO*_2_ is the total amount of ^14^C recovered in the NaOH traps (kBq); and ^14^*Cs* is the ^14^C recovered in the 0.5 M K_2_SO_4_ soil extract (kBq).

Microbial carbon use efficiency (^14^*CUE*) was calculated using Equation (2):

(2) C14UE=C14iC14i+C14O2

### 2.3. Laboratory Assay—^33^P

Secondly, for the ^33^P assay, 20 replicates of soil samples (1 g) were placed in individual sterile 50 mL polypropylene centrifuge tubes. Subsequently, ^33^P-H_2_O (25 µL; 289.2 kBq mL^−1^) was applied to the soil. After 24 h ^33^P-equilibrating, the soil was submitted to the dilution-to-extinction methodology to generate the same five soil microbiomes, as described above, using 50 µL of soil suspension. Subsequently, the treatments were incubated [room temperature (20 ± 5 °C), 14 d] and then subjected to a sequential ^33^P chemical fractionation [18], as briefly described. The soil was first shaken (200 rpm; 16 h) with deionized water (30 mL) in the presence of a capsule containing a mixed cation–anion exchange resin (Unibest International, Kennewick, WA, Australia). Afterward, ^33^P was desorbed from the resin by placing the capsule in 30 mL of 0.5 M HCl for 16 h (^33^P_AER_). The soil was then centrifuged (3850× *g*; 30 min), and the water was discarded. The remaining soil was sequentially extracted with 0.5 M NaHCO_3_ (30 mL; 200 rpm; 16 h), centrifuged (3850× *g*; 30 min), and the supernatant recovered (^33^P_NaHCO3_). This process was repeated with the following extractants: 0.1 M NaOH, 1 M HCl, and 0.5 M NaOH, for ^33^P_0.1NaOH_, ^33^P_HCl_, and ^33^P_0.5NaOH_, respectively. After all the extractions, the remaining soil was dried (40 °C, 48 h), ground, and digested (0.1 g) with perchloric acid (10 mL, 200 °C, 4 h), which represented the residual P (^33^P_Res_). ^33^P activity for each fraction was determined by using the extractant (1 mL) and Optiphase HiSafe 3 scintillation cocktail (4 mL) in a liquid scintillation counter. Afterward, ^33^P-data were corrected according to ^33^P-decay (ca. 25 d) [19] back to the point at which the tracer was added to the soil by using Equation (3):

(3) $$P_{o}=\frac{P}{e^{-\lambda t}}$$

where *P_o_* is the ^33^P-activity decay corrected to the point when the tracer was added to the soil (kBq); *P* is the ^33^P-activity at time of counting (kBq); *t* is the time between tracer addition and measurement (d); *e* = 2.71828; and *λ* is the ^33^P-decay constant (*λ* = 0.0273539).

Then, the ^33^P fractions were grouped according to the lability as follows: (i) labile ^33^P (^33^P_AER_ and ^33^P_NaHCO3_); (ii) moderately labile (^33^P_0.1NaOH_ and ^33^P_HCl_); and (iii) non-labile (^33^P_0.5NaOH_ and ^33^P_Res_).

### 2.4. Statistical Analysis

Data were separated into two data sets according to the land use (forest and cultivated area). For ^14^CO_2_ modeling, Equation (4) was used:

(4) $$y=\frac{K}{1\;+\;e^{-r\left(t-b\right)’}}$$

where *K* represents the cumulative frequency at the upper limit (maximum value that the function can reach as time increases; *r* controls the speed at which the curve grows (higher values indicate a more abrupt transition between the initial and final states, making the curve steeper); t represents the time at which the fastest transition occurs between the accelerated and decelerated growth states (*t* → ∞; however, in this study, *t* is limited by the maximum respiration rate that the environment can sustain); *b* determines the location of the peak on the x-axis (in this case, *t*).

Parameter estimation was performed using a Bayesian approach, considering non-informative prior distributions, through Hamiltonian Monte Carlo (HMC) sampling. The results of the estimation of the nonlinear model parameters (Equation (4)) were obtained for each soil type and their respective microbial manipulations. To support the analyses, the Bayesian regression models—brms package—were implemented in the R software (version 4.5.1). Informative priors were specified to reflect the response scale: *K*∼Normal (100, 10), *r*∼Normal (1, 0.5), and *b*∼Normal (5, 1). Sampling was performed using four chains (iter = 4000, warmup = 1000, chains = 4). Convergence was evaluated through the Gelman–Rubin potential scale reduction factor $\hat{R}$, effective sample sizes, and visual inspection of trace plots. All $\hat{R}$ values were ≤1.001, and no divergent transitions were detected.

Additionally, one-way analysis of variance (ANOVA) was performed considering microbiome manipulation as a fixed factor within the generalized linear model (GLM). Data distribution of all variables was also checked. Tukey’s post hoc test at the 5% level of significance was used to perform comparisons of the means. The one-way analysis was performed using SAS 9.4 software.

## 3. Results

### 3.1. Microbial Activity Characterization—^14^C Approach

Based on the parameters analyzed (Equation (4)), the maximum ^14^CO_2_-respiration capacity (*K*), growth rate (*r*), and transition time (*b*) were estimated for each soil type and their respective microbial manipulations (Table 2 and Table 3).

**Figure 2:**
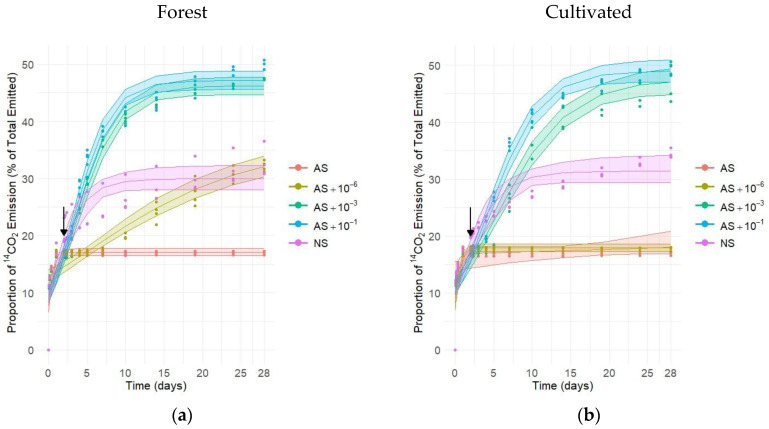
^14^CO_2_ emission over 28 days of incubation and after application of ^14^C-labeled glucose to soil, followed by soil microbiome manipulation (↓ arrow indicates the manipulation event). Treatments included the following: AS (autoclaved soil, 121 °C, 103 kPa, 1 h); AS with microbial inoculation at dilutions of 10^−6^, 10^−3^, and 10^−1^; and NS (non-autoclaved soil, no manipulation for soils sampled under (**a**) forest and (**b**) cultivated soil).

Regarding the maximum respiration (*K*), for both soil types, there was no difference between AS + 10^−1^ and AS + 10^−3^. In forest soil, AS + 10^−1^ and AS + 10^−3^ reached ^14^C-respiration capacity (*K*) of 49.01% and 47.49%, respectively (Figure 2a). For this soil, no significant differences were observed between the AS + 10^−6^, AS, and NS treatments, and all treatments presented maximum ^14^CO_2_ emissions (*K*) below 35%. For respiration growth rate (*b*), for forest soil, AS + 10^−6^ and AS treatments presented high standard errors, resulting in wide credibility intervals, which makes it difficult to interpret the differences between treatments regarding growth rate. For logistic curve transition time (*b*), for forest soil, no significant differences were observed between the AS + 10^−1^ and AS + 10^−3^ treatments, both presenting the longest transition times, with values of 4.37 and 5.42, respectively. On the other hand, NS treatment showed a transition time of 1.41. Again, in the forest soil, the AS + 10^−6^ and AS treatments presented high standard errors regarding transition time, resulting in wide credibility intervals, which makes it difficult to interpret the differences between treatments.

For cultivated soil, the AS + 10^−1^ and AS + 10^−3^ treatments presented maximum CO_2_ emissions (*K)* of 47.11% and 46.19%, respectively (Figure 2b). AS treatment showed the highest microbial growth rate (*r*) (2.56), while the AS + 10^−6^ treatment exhibited the lowest rate (*r*) (0.101). No significant differences were observed between the AS + 10^−1^ (0.41), AS + 10^−3^ (0.36), and NS (0.55) treatments regarding growth rate (*r*). Significant differences were observed in the transition time (*b*) between treatments. AS + 10^−6^ treatment presented the longest transition time (*b*) (5.65), while the lowest value was observed in the AS treatment (0.08).

**Figure 3:**
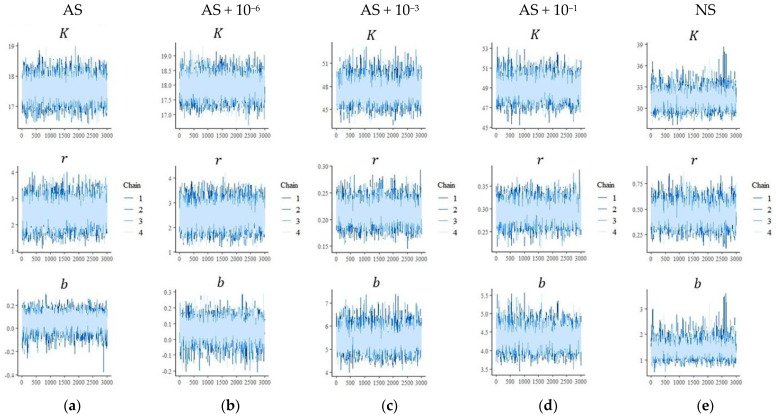
Trace plots of the Markov Chain Monte Carlo (MCMC) chains for the Bayesian parameters *K*, *r*, and *b* describing ^14^C dynamics after the application of ^14^C-labeled glucose to soil. Five manipulated microbial soil communities were obtained: (**a**) AS, autoclaved soil (121 °C, 103 kPa, 1 h) followed by microbial inoculation of dilutions; (**b**) AS + 10^−6^; (**c**) AS + 10^−3^; (**d**) AS + 10^−1^; and (**e**) NS, non-autoclaved soil without manipulation, soil sampled under a forest area.

**Figure 4:**
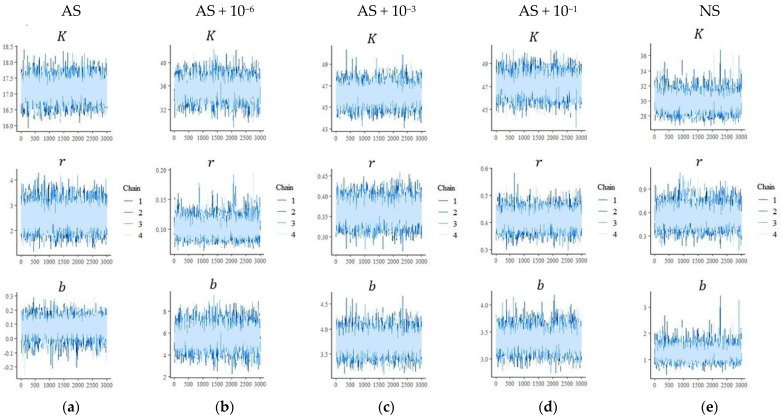
Trace plots of the Markov Chain Monte Carlo (MCMC) chains for the Bayesian parameters *K*, *r*, and *b* describing ^14^C dynamics after the application of ^14^C-labeled glucose to soil. Five manipulated microbial soil communities were obtained: (**a**) AS, autoclaved soil (121 °C, 103 kPa, 1 h) followed by microbial inoculation of dilutions; (**b**) AS + 10^−6^; (**c**) AS + 10^−3^; (**d**) AS + 10^−1^; and (**e**) NS, non-autoclaved soil without manipulation, soil sampled under a cultivated area.

The MCMC chains show stable behavior and clear signs of convergence for the parameters *K*, *r*, and *b* (Figure 3 and Figure 4). These results correspond to the parameters presented in Table 2 and Table 3.

**Figure 5:**
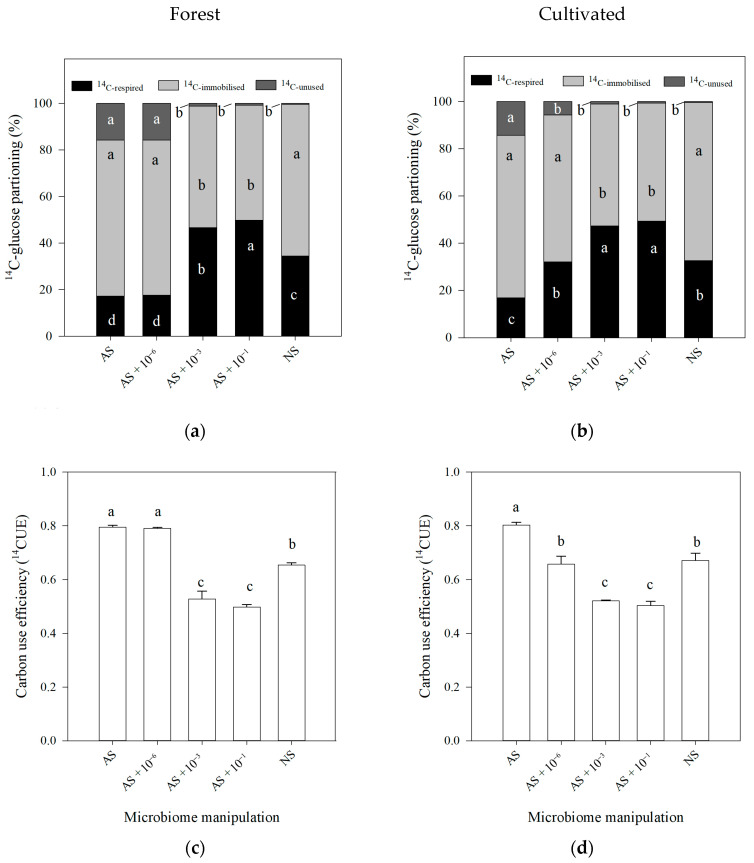
(**a**,**b**) ^14^C partitioning into respired, immobilized, and unused fractions; (**c**,**d**) ^14^C use efficiency after 28 days of incubation and after application of ^14^C-labeled glucose to soil, followed by soil microbiome manipulation. Treatments included the following: AS (autoclaved soil, 121 °C, 103 kPa, 1 h); AS with microbial inoculation at dilutions of 10^−6^, 10^−3^, and 10^−1^; and NS (non-autoclaved soil, no manipulation for soils sampled under (**a**–**c**) forest and (**b**–**d**) cultivated soil). Common letters indicate no significant differences according to Tukey’s post hoc test (*p* ≤ 0.05). Error bars represent 95% confidence intervals.

Considering the ^14^CO_2_ evolved during the micro-organism’s respiration, immobilized in the micro-organism’s biomass, and the unused ^14^C, in general, the tendency according to the manipulated microbial community in the distribution was similar for both forest and cultivated soils (Figure 5a,b). According to the ^14^C partition, under AS, the lowest release of ^14^CO_2_-respirated was observed among the treatments, with high immobilization in the microbial biomass (i.e., anabolism) and the highest ^14^C-unused. When a low number of cells was reincorporated into the sterile soil (AS + 10^−6^), for forest soil, a very similar trend was observed in comparison to AS; for cultivated soil, under AS + 10^−6^, an increase in the ^14^C-respirated was observed, with a reduction in the ^14^C-unused. When a high number of cells were reincorporated in the sterile soil (AS + 10^−3^ and AS + 10^−1^), regardless of the land use, the highest ^14^C-respirated was observed, with intermediate content of ^14^C-immobilized and low ^14^C-unused. For NS treatment, intermediary emissions of ^14^CO_2_, with high ^14^C-immobilization and low ^14^C-unused, were observed.

In terms of C use efficiency (CUE), for both forest and cultivated soils, the highest values were observed in the AS (Figure 5c,d). For the forest soil, the treatment AS + 10^−6^ also presented high values of ^14^CUE. For both forest and cultivated soil, the lowest ^14^CUE was observed under AS + 10^−1^ and AS + 10^−3^, and intermediate values of ^14^CUE were observed under NS.

### 3.2. N Dynamics in Autoclaved Soil with the Levels of Microbial Reinoculation

**Figure 6:**
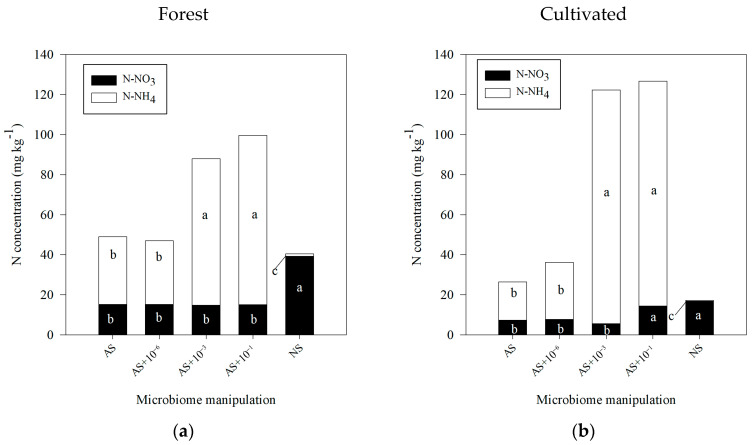
Nitrogen (N-NO_3_ and N-NH_4_ concentrations) after 28 days of incubation and after application of ^14^C-labeled glucose to soil, followed by soil microbiome manipulation. Treatments included the following: AS (autoclaved soil, 121 °C, 103 kPa, 1 h); AS with microbial inoculation at dilutions of 10^−6^, 10^−3^, and 10^−1^; and NS (non-autoclaved soil, no manipulation for soils sampled under (**a**) forest and (**b**) cultivated soil). Common letters indicate no significant differences according to Tukey’s post hoc test (*p* ≤ 0.05).

Regarding the N-inorganic forms, in general, for both forest and cultivated soils, the autoclaving procedure, either with or without reinoculation, promoted considerable changes in the nitrate (N-NO_3_) and ammonium (N-NH_4_) proportions in the soil (Figure 6).

In forest soil, under the NS condition (without microbial manipulation), nitrate (N-NO_3_^−^) and ammonium (N-NH_4_^+^) contents were 39.23 mg kg^−1^ and 1.26 mg kg^−1^, respectively (Figure 6a). Autoclaving reduced N-NO_3_^−^ by about half (average ≈ 15 mg kg^−1^) and increased N-NH_4_^+^ across all manipulated conditions compared to NS. In sterile soil (AS) and AS + 10^−6^ (low cell reinoculation), intermediate N-NH_4_^+^ values were observed, with ~30-fold increases (AS = 33.77 mg kg^−1^; AS + 10^−6^ = 31.76 mg kg^−1^). In contrast, reinoculation with higher cell numbers (AS + 10^−3^ and AS + 10^−1^) resulted in the highest N-NH_4_^+^ concentrations (AS + 10^−3^ = 73.06 mg kg^−1^; AS + 10^−1^ = 84.50 mg kg^−1^).

In cultivated soil, the NS condition showed 16.82 mg kg^−1^ of N-NO_3_^−^ and 0.45 mg kg^−1^ of N-NH_4_^+^. Autoclaving and reinoculation with low or intermediate cell numbers (AS, AS + 10^−6^, and AS + 10^−3^) reduced N-NO_3_^−^ by ~5-fold (AS = 7.28 mg kg^−1^; AS + 10^−6^ = 7.67 mg kg^−1^; AS + 10^−3^ = 5.56 mg kg^−1^), whereas AS + 10^−1^ showed no difference compared to NS (14.34 mg kg^−1^). For N-NH_4_^+^, cultivated soil exhibited ~20-fold increases in AS and AS + 10^−6^ (AS = 19.18 mg kg^−1^; AS + 10^−6^ = 28.45 mg kg^−1^) compared to NS. Reinoculation with high cell numbers produced the largest increases, with N-NH_4_^+^ concentrations exceeding 100-fold (AS + 10^−3^ = 116.83 mg kg^−1^; AS + 10^−1^ = 112.40 mg kg^−1^).

### 3.3. P Dynamics in Autoclaved Soil with Levels of Microbial Reinoculation

**Figure 7:**
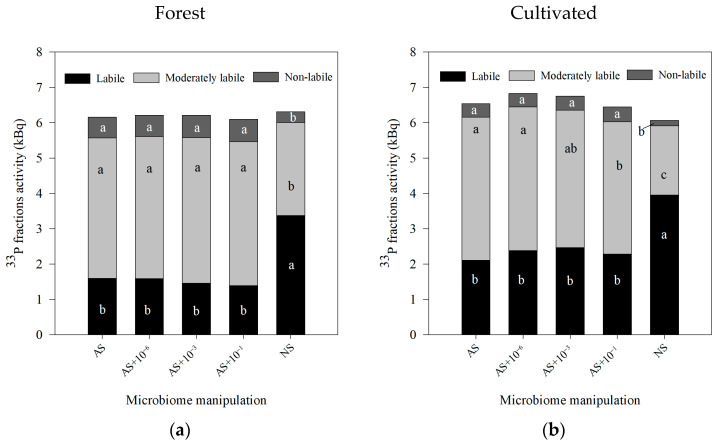
^33^P soil fractionation * after 14 days of incubation and after application of ^33^P labilled to soil, followed by soil microbiome manipulation. Treatments included the following: AS (autoclaved soil, 121 °C, 103 kPa, 1 h); AS with microbial inoculation at dilutions of 10^−1^, 10^−3^, and 10^−6^; and NS (non-autoclaved soil, no manipulation for soils sampled under (**a**) forest and (**b**) cultivated soil). Common letters indicate no significant differences according to Tukey’s post hoc test (*p* ≤ 0.05). * ^33^P soil fractionation: labile ^33^P (^33^P_-AER_ and ^33^P_NaHCO3_); moderately labile (^33^P_0.1_NaOH_ and ^33^P_HCl_); and non-labile (^33^P_0.5_NaOH_ and ^33^P_Res_), according to Hedley et al. [18].

Regardless of the land use, for both forest and cultivated soil, ^33^P activity showed the same trend for the P lability (Figure 7). Higher ^33^P-labile was observed in NS, and a reduction in the autoclaved soil (AS), either with or without dilution inoculations. Hence, an increase in the ^33^P-moderately labile and ^33^P-non labile activity was observed (Figure 7).

For forest, natural soil (NS) without autoclaving showed the highest labile fractions ^33^P-activity (^33^P_AER_ and ^33^P_NaHCO3_; Table 4). Considering only the ^33^P_AER_, the treatments AS, without reinoculation, and AS + 10^−6^, with the smallest reinoculation, showed intermediate ^33^P activity, and when the sterile soil was reinoculated with a higher number of cells (AS + 10^−3^ and AS + 10^−1^), the lowest activity was observed (Table 4). Considering the ^33^P-moderate labile fractions (^33^P_0.1NaOH_ and ^33^P_HCl_), an increment in the ^33^P activity was observed for the autoclaved soils, regardless of the reinoculation level. For non-labile fractions, the lowest ^33^P activity was observed in the ^33^P_0.5NaOH_ fraction in NS, and no difference was observed in ^33^P_Res_.

For the cultivated area, for labile ^33^P fractions, the highest ^33^P_AER_ was observed in the NS, but no differences for the sterile soil, regardless of the reinoculation, were observed (Table 5), and no effect on the ^33^P_NaHCO3_ activity was observed among all the treatments. For the ^33^P-moderated labile and ^33^P-non labile fractions, NS showed lower activity in comparison to the sterile autoclaved soil (AS).

## 4. Discussion

In general, the results from both forest and cultivated soils presented the same trend after autoclaving treatment for ^14^C, N, and ^33^P dynamics, with particularities for ^14^CO_2_ and N-NO_3_.

### 4.1. ^14^C and N Findings According to the Soil Manipulation Using Autoclaving

Regarding microbial activity as a response to microbial respiration (^14^CO_2_ emission), for both forest and cultivated soils, the non-autoclaved soil (NS) showed a constant increment in the ^14^CO_2_ emission over time, and no abrupt changes in the respiration. However, after the autoclaving procedure in the labeled soil (^14^C-glucose application), a reduction in the microbial activity was observed. For both soils, the treatments using autoclaved soil (AS), without inoculation, did not show emission of ^14^CO_2_ compared to the NS, indicating a lack of microbial activity, even after the application of a ^14^C easily degradable source, such as glucose, which is the most common and abundant product released in the rhizosphere [20]. Thus, unlike the molecular analysis, DNA sequencing was used by Arruda et al. [11] to characterize the microbial community after autoclaving, and they found no differences between non-autoclaved and autoclaved soil, but had an opposite response in ^14^CO_2_ respiration between such treatments for microbial activity.

After autoclaving, the reinoculation, with a small number of cells (AS + 10^−6^), had notable differences between the soils from the forest and cultivated areas. In the forest soil, the AS + 10^−6^ treatment followed the same trend as AS, with very low respiration. This may indicate that the organisms reintroduced into the AS were not able to promote changes in the ^14^CO_2_-respiration. In contrast, in the cultivated soil, an increase in ^14^CO_2_-respiration was observed under AS + 10^−6^, reaching values close to those of NS. This may be because agricultural practices have selected organisms that, even when reintroduced into the AS in low amounts, were able to sustain high respiration rates.

When both tested soils were autoclaved and inoculated with a high number of cells (AS + 10^−1^ and AS + 10^−3^), an abrupt ^14^CO_2_ emission was observed the following day after autoclaving and reinoculation, corroborating the beneficial effect of reinoculation with high concentrations of micro-organisms. In cultivated soil, the other treatments showed significant differences, although all exhibited maximum CO_2_ emissions below 40%. The inoculation after ^14^C-glucose application found an environment very rich in ^14^C-glucose, which triggered the apparent priming effect in micro-organisms during the first two weeks, corroborating Pascault et al. [21]. These authors stimulated the soil priming effect of different functional groups of bacteria by using various plant residues. They attributed this rapid initial consumption in the first two weeks to the presence of easily degradable compounds. This rapid usage of ^14^C is performed by the r-strategist, or copiotrophs. Once the labile C source is over, these organisms die or become dormant [22,23], and the establishment of the basal activity occurs, and then we see a stabilization in ^14^CO_2_ emission.

To compare the decomposers’ C metabolism among treatments, the C use efficiency (^14^CUE) [24] was used. For the forest, the highest values of ^14^CUE were observed under AS and AS + 10^−6^ due to the low release of ^14^CO_2_, indicating that the C is not being lost to the atmosphere by respiration, as no microbial activity is observed in these treatments. For cultivated soil, this effect was observed only in AS. The opposite was observed under AS + 10^−1^ and AS + 10^−3^, where high ^14^CO_2_ was lost to the atmosphere and less ^14^C was immobilized in biomass from the easily degradable ^14^C-glucose.

Soil autoclaving typically causes a shift from NO_3_^−^ to NH_4_^+^ by disrupting microbial N processes. Steam sterilization at high temperatures eliminates nitrifying bacteria, which normally oxidize NH_4_^+^ to NO_3_^−^, halting nitrification and causing NH_4_^+^ to accumulate while NO_3_^−^ declines [25,26]. The death of microbes also releases organic N as NH_4_^+^ via thermal mineralization, further raising NH_4_^+^-N concentration [26,27]. In addition, any residual NO_3_^−^ can be converted to NH_4_^+^ via dissimilatory nitrate reduction to ammonium by surviving or reintroduced microbes [28], thereby retaining N as NH_4_^+^ rather than losing it as leachable NO_3_^−^. These mechanisms explain why AS became NH_4_^+^-rich and NO_3_^−^-poor after autoclaving.

The comparison between forest and cultivated soils demonstrates how microbial diversity and land use history modulate the shift from NO_3_^−^ to NH_4_^+^. Forest soil initially contained more NO_3_^−^-N (and less NH_4_^+^-N), owing to abundant and diverse nitrifiers, whereas agricultural disturbance lowers microbial diversity and nitrifier abundance, yielding reduced baseline NO_3_^−^ [25,29]. Thus, autoclaving forest soil causes a larger absolute drop in NO_3_^−^-N than an already NO_3_^−^-poor cultivated soil. Accordingly, in a recent study, all autoclaved forest soil treatments remained much lower in NO_3_^−^-N than the native control, whereas in cultivated soil, the highest reinoculation restored NO_3_^−^-N levels to control levels [30]. Both soil types showed sharp rises in NH_4_^+^ after sterilization, but the low initial NH_4_^+^-N concentration in forest soil meant a proportionally greater increase. Overall, nitrification in the forest soil was harder to revive, perhaps due to reliance on specialized nitrifiers that were eliminated.

Microbial diversity and functional redundancy are critical in recovery. Nitrification is carried out by a few specialized taxa that are highly sensitive to perturbation [25]; notably, nitrifiers often form only a minor fraction (~10^4^–10^6^ cells g^−1^) of the community, making them easily lost [30]. Their removal severely inhibits NO_3_^−^ production. However, if a sufficiently diverse community is reintroduced, nitrification can rebound due to functional redundancy (i.e., the presence of multiple species capable of performing the same process) [30]. Experiments confirm that strong reinoculation (high biomass and diversity inoculate) can rescue nitrification in AS, whereas low diversity inoculate often fails to restore this function [30]. Therefore, reinoculation in AS intensity governs N-cycle recovery: minimal inoculate leaves NH_4_^+^-N concentration elevated and NO_3_^−^-N depressed, while rich inoculate re-establishes nitrification, especially in cultivated soil.

### 4.2. ^33^P Findings According to the Soil Manipulation Using Autoclaving

Regarding ^33^P dynamics, the ^33^P fractionation indicated that the autoclaving reduced the labile ^33^P compared to the NS, for both soils. This was related to the biological effect on the soil P availability, reducing the processes such as mineralization of the soil organic matter through the exudation of enzymes, such as phytases and phosphatases, acid, and alkaline. After the autoclaving procedure, the biodiversity is reduced, and due to the lack of biological processes, the P availability is reduced. Our results indicated that the reinoculation in the sterile soil did not result in changes in the P lability. Additionally, a massive increase in ^33^P_HCl_ after autoclaving was observed in comparison to the NS. This ^33^P_HCl_ fraction corresponds to minerals of the soil containing P-Ca, such as apatite. These results may indicate that autoclaving, combining high temperature and pressure, was able to promote rearrangements in the P forms, favoring the link between P-Ca. Together, those results indicate that the changes observed in the ^33^P fraction distribution after autoclaving were due to the procedure itself, but the reinoculation of the micro-organisms did not contribute to any change.

Unlike the N cycle, which is very dynamic with rapid transformation processes, the P biodynamics in the soil are very slow. Therefore, the autoclaving strongly harmed the biological process, affecting the P dynamics, and even after reinoculation of organisms in the system, no recovery response was observed.

### 4.3. Limitations and Outlooks

To investigate soil microbial manipulation, experiments under controlled conditions were required. However, this represents a limitation for the representativeness of field conditions. To ensure control, 5 g of soil was used in the laboratory assays with ^14^C and N, and 1 g of soil for the ^33^P assays. To preserve field representativity, soil samples were sieved at 5 mm to maintain aggregates and structure without compromising experimental control.

To prevent contamination by airborne micro-organisms, incubation tubes were kept closed, which may have created a microclimate inside. Nevertheless, 50 mL tubes were used to allow a micro-atmosphere more representative of field conditions. Considering these aspects, the incubation period was relatively short. However, in the ^14^C and N assays, CO_2_ respiration reached stability across treatments, indicating that the period was sufficient. In the ^33^P assays, the isotope half-life limits longer experiments; yet the high recovery of ^33^P across treatments confirmed that the timeframe was also adequate.

Another limitation concerns the autoclaving procedure itself. Because sterilization involves high temperature and pressure, chemical changes in the soil may occur. To account for this, negative (AS) and positive (NS) controls were included for comparison with reinoculated treatments. The AS control reflects the autoclaving effect without a microbial community, whereas NS represents the undisturbed community without autoclaving. By comparing these with reinoculated soils, it was possible to assess how different levels of micro-organisms recolonize sterile soil.

### 4.4. Highlighting the Broader Implications of Microbial Manipulation Experiments

The first step is to recognize the limitations of the different methods available to promote changes in soil microbial communities, as the choice of method is critical. Once the method is selected, reliable indicators of microbial activity are needed. Arruda et al. [11] showed that sequencing data did not provide consistent evidence of microbial change after autoclaving, whereas in the present study, ^14^CO_2_ respiration proved to be a robust indicator of microbial activity after the autoclaving procedure. Results showed that reinoculation of autoclaved soil with high microbial richness increased activity to levels even higher than those of non-autoclaved soil (NS), while microbial activity in autoclaved soil without reinoculation (AS) was null. These findings highlight the potential of this approach to reproduce outcomes under contrasting soil-management scenarios, with extreme degradation and biodiversity levels. Thus, this methodology represents a relevant advance in soil ecology.

Beyond microbial activity measurements, it is also important to assess changes in nutrient dynamics, as the loss of biodiversity may affect key soil functions such as N and P dynamics. In this study, high reinoculation levels (AS + 10^−3^ and AS + 10^−1^) decreased N-NO_3_^−^ and increased N-NH_4_^+^ compared with undisturbed soil (NS), whereas no reinoculation or low reinoculation (AS + 10^−6^) also increased N-NH_4_^+^ values, but in a lower range. These patterns suggest that microbial activity, rather than the autoclaving procedure itself, was the main driver of N transformations. In contrast, autoclaving strongly reduced the size of labile ^33^P fractions, and reinoculation did not alter ^33^P lability during the 14-day incubation, indicating that for P, chemical changes caused by autoclaving had a greater influence than microbial activity.

Overall, the laboratory results demonstrated that autoclaving effectively generated a gradient of microbial activity. For future experiments involving plants, the AS treatment may be unnecessary, as its effects are already well characterized in this study. Among the reinoculation levels tested (10^−1^, 10^−3^, and 10^−6^), the results suggest that 10^−3^ may be the most informative. Treatments at 10^−1^ produced responses similar to undisturbed soil (NS), while 10^−6^ mimicked near-sterile conditions, which are unrealistic for sustainable land management. Therefore, the 10^−3^ level may serve as a proxy for regenerative agriculture, representing an intermediate state between highly degraded and undisturbed soils.

## 5. Conclusions

In conclusion, (i) the ^14^CO_2_ emission from rich microbial reinoculation in autoclaved soil increased microbial activity to levels higher than in non-autoclaved soil (NS). In contrast, microbial activity in autoclaved soil without reinoculation (AS) was null; and (ii) regarding the N dynamics, in autoclaved soils, the microbial levels increased N-NH_4_^+^, with an evident increase in the AS + 10^−3^ and AS + 10^−1^, and reduced N-NO_3_^−^ contents in comparison to the NS. For P, the autoclaving procedure itself reduced the ^33^P lability, regardless of the levels of microbial community reinoculated.

## Figures and Tables

**Table 1:** Chemical analysis of Eutric Cambisol [13] sampled in Wales (UK), 0–10 cm depth, under forest and cultivated area (maize), before the onset of the laboratory experiment. Values represent means (*n* = 4) of four replicates; ± represents the standard error of the mean (*n* = 4) of four replicates.

Land Use	pH	P	K	Mg	Ca	S	Mn	Cu	B	Zn	Mo	Fe	CEC *
		ppm											meq/100 g
Forest	5.4 ± 0.1	17 ± 1	87 ± 2	133 ± 2	1149 ± 27	5.0 ± 0.0	87 ± 8	7.2 ± 0.1	0.74 ± 0.04	7.2 ± 0.2	0.08 ± 0.02	784 ± 46	11.0 ± 0.5
Cultivated	6.4 ± 0.1	36 ± 1	74 ± 3	70 ± 2	1851 ± 96	4.0 ± 0.0	94 ± 6	9.6 ± 0.5	0.97 ± 0.05	7.2 ± 0.2	0.08 ± 0.01	854 ± 21	11.3 ± 0.5

* CEC: cation exchange capacity.

**Table 2:** Estimation of the *K*, *r,* and *b* parameters and the corresponding lower (LL) and upper (UL) limits of the 95% credible interval and potential scale reduction $\hat{R}$ of 28 days incubation and after application of ^14^C-labeled glucose to soil, followed by soil microbiome manipulation. Treatments included the following: AS (autoclaved soil, 121 °C, 103 kPa, 1 h); AS with microbial inoculation at dilutions of 10^−6^, 10^−3^, and 10^−1^; and NS (non-autoclaved soil, no manipulation for soils sampled under forest soil).

Microbiome Manipulation	Estimate	LL 2.50%	UL 97.50%	$\hat{R}$
	*K* parameter			
AS	17.57 b	16.92	18.24	1.00
AS + 10^−6^	21.41 b	17.33	33.23	1.00
AS + 10^−3^	47.49 a	45.11	50.10	1.00
AS + 10^−1^	49.01 a	47.17	51.00	1.00
NS	31.35 b	29.31	33.98	1.00
	r parameter			
AS	2.45a	1.65	3.24	1.00
AS + 10^−6^	1.88 ab	0.01	3.29	1.00
AS + 10^−3^	0.21 b	0.18	0.25	1.00
AS + 10^−1^	0.29 ab	0.25	0.34	1.00
NS	0.45 a	0.26	0.65	1.00
	b parameter			
AS	0.06 ab	−0.07	0.17	1.00
AS + 10^−6^	1.33 ab	−0.06	6.41	1.00
AS + 10^−3^	5.42 a	4.65	6.33	1.00
AS + 10^−1^	4.37 a	3.88	4.91	1.00
NS	1.41 b	0.95	2.08	1.00

Common letters do not indicate differences by credible interval.

**Table 3:** Estimate of the *K*, *r*, and *b* parameters and the corresponding lower (LL) and upper (UL) limits of the 95% credible interval and potential scale reduction $\hat{R}$ of 28 days incubation and after application of ^14^C-labeled glucose to soil, followed by soil microbiome manipulation. Treatments included the following: AS (autoclaved soil, 121 °C, 103 kPa, 1 h); AS with microbial inoculation at dilutions of 10^−6^, 10^−3^, and 10^−1^; and NS (non-autoclaved soil, no manipulation for soils sampled under cultivated soil).

Microbiome Manipulation	Estimate	LL 2.50%	UL 97.50%	$\hat{R}$
	*K* parameter			
AS	17.12 d	16.53	17.74	1.00
AS + 10^−6^	35.52 b	32.38	38.66	1.00
AS + 10^−3^	46.19 a	44.67	47.83	1.00
AS + 10^−1^	47.11 a	45.50	48.77	1.00
NS	29.90 c	27.96	32.30	1.00
	r parameter			
AS	2.56 a	1.76	3.42	1.00
AS + 10^−6^	0.10 c	0.08	0.13	1.00
AS + 10^−3^	0.36 b	0.31	0.41	1.00
AS + 10^−1^	0.41 b	0.35	0.48	1.00
NS	0.55 b	0.33	0.80	1.00
	b parameter			
AS	0.08 d	−0.04	0.18	1.00
AS + 10^−6^	5.65 a	3.82	7.51	1.00
AS + 10^−3^	3.73 ab	3.38	4.14	1.00
AS + 10^−1^	3.35 b	3.02	3.72	1.00
NS	1.25 c	0.83	1.78	1.00

Common letters do not indicate differences by credible interval.

**Table 4:** ^33^P soil fractionation * after 14 days of incubation and after application of ^33^P labilled to soil, followed by soil microbiome manipulation. Treatments included the following: AS (autoclaved soil, 121 °C, 103 kPa, 1 h); AS with microbial inoculation at dilutions of 10^−1^, 10^−3^, and 10^−6^; and NS (non-autoclaved soil, no manipulation for soils sampled under forest soil). Values represent mean ± represent the standard error of the mean (*n* = 4).

Soil Microbiome Manipulation	^33^P_AER_	^33^P_NaHCO3_	^33^P_0.1NaOH_	^33^P_HCl_	^33^P_0.5NaOH_	^33^P_Res_	^33^P_Total_
	kBq						
AS	0.98 ± 0.04 b	0.61 ± 0.03 b	2.97 ± 0.04 a	1.01 ± 0.05 a	0.51 ± 0.02 a	0.08 ± 0.00 ns	6.16 ± 0.08
AS + 10^−6^	1.00 ± 0.06 b	0.59 ± 0.02 b	3.02 ± 0.06 a	1.01 ± 0.03 a	0.52 ± 0.02 a	0.09 ± 0.00	6.21 ± 0.16
AS + 10^−3^	0.85 ± 0.06 c	0.61 ± 0.02 b	3.08 ± 0.06 a	1.05 ± 0.05 a	0.55 ± 0.02 a	0.08 ± 0.01	6.21 ± 0.07
AS + 10^−1^	0.80 ± 0.03 c	0.59 ± 0.04 b	3.03 ± 0.19 a	1.04 ± 0.04 a	0.55 ± 0.01 a	0.09 ± 0.01	6.10 ± 0.25
NS	2.14 ± 0.13 a	1.23 ± 0.13 a	2.34 ± 0.14 b	0.30 ± 0.02 b	0.26 ± 0.03 b	0.03 ± 0.00	6.31 ± 0.41

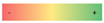
 Means followed by the same letter are not significantly different according to Tukey’s test (*p* < 0.05). Different letters indicate significant differences among treatments. ns means not significant. Color scale was used for all sets of data for each variable. * ^33^P soil fractionation was determined according to Hedley et al. [18].

**Table 5:** ^33^P soil fractionation * after 14 days of incubation and after application of ^33^P labilled to soil, followed by soil microbiome manipulation. Treatments included the following: AS (autoclaved soil, 121 °C, 103 kPa, 1 h); AS with microbial inoculation at dilutions of 10^−1^, 10^−3^, and 10^−6^; and NS (non-autoclaved soil, no manipulation for soils sampled under cultivated soil). Values represent mean ± represent the standard error of the mean (*n* = 4).

Soil Microbiome Manipulation	^33^P_AER_	^33^P_NaHCO3_	^33^P_0.1NaOH_	^33^P_HCl_	^33^P_0.5NaOH_	^33^P_Res_	^33^P_Total_
	kBq						
AS	1.51 ± 0.05 b	0.59 ± 0.02 ns	3.23 ± 0.10 a	0.82 ± 0.03 ab	0.34 ± 0.02 a	0.05 ± 0.01 ns	6.54 ± 0.15
AS + 10^−6^	1.57 ± 0.12 b	0.81 ± 0.27	3.23 ± 0.11 a	0.84 ± 0.07 a	0.34 ± 0.03 a	0.05 ± 0.00	6.83 ± 0.23
AS + 10^−3^	1.66 ± 0.07 b	0.80 ± 0.29	3.15 ± 0.13 ab	0.74 ± 0.02 b	0.34 ± 0.02 a	0.05 ± 0.01	6.75 ± 0.33
AS + 10^−1^	1.70 ± 0.23 b	0.58 ± 0.02	2.98 ± 0.16 b	0.77 ± 0.04 ab	0.37 ± 0.04 a	0.05 ± 0.01	6.45 ± 0.04
NS	3.37 ± 0.13 a	0.58 ± 0.03	1.79 ± 0.04 c	0.18 ± 0.01 c	0.12 ± 0.01 b	0.02 ± 0.00	6.06 ± 0.11

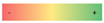
 Means followed by the same letter are not significantly different according to Tukey’s test (*p* < 0.05). Different letters indicate significant differences among treatments. ns means not significant. Color scale was used for all sets of data for each variable. * ^33^P soil fractionation was determined according to Hedley et al. [18].

## Data Availability

The original contributions presented in this study are included in the article. Further inquiries can be directed to the corresponding author.

## Abbreviations

The following abbreviations are used in this manuscript:

NS	Non-autoclaved soil
AS	Autoclaved soil
AS + 10^−1^	Autoclaved soil + wv^−1^ of NS added to the AS
AS + 10^−3^	Autoclaved soil + wv^−1^ of NS added to the AS
AS + 10^−6^	Autoclaved soil + wv^−1^ of NS added to the AS
